# Sharing is caring: a review on Oxytocin role in human behaviour and clinical implications

**DOI:** 10.1192/j.eurpsy.2022.1898

**Published:** 2022-09-01

**Authors:** F. Ramalheira, M. Conde Moreno

**Affiliations:** 1 Centro hospitalar Psiquiátrico de Lisboa, Serviço De Electroconvulsoterapia, Lisboa, Portugal; 2 Centro hospitalar Psiquiátrico de Lisboa, Hospital De Dia, Lisboa, Portugal

**Keywords:** Oxytocin in humans, behaviour, psychiatry, Oxytocin

## Abstract

**Introduction:**

Oxytocin, also known as the love molecule, was discovered in 1906 because of its effects on uterine contractions. It exists in all mammals and is partly responsible for delivery. Nonetheless, it seems that oxytoxin also takes part in something as important to nursing as the physical changes in childbirth – the behavioural predisposition to form human bonds and to care for others.

**Objectives:**

Present a review on Oxytocin and its functions in human behaviour and possible clinical implications

**Methods:**

Pubmed and Google Scholar search using the keywords “oxytocin”, “behaviour”, “oxytocin in humans” and “psychiatry”.

**Results:**

Besides acting as a peripheral hormone following posterior hypophisis secretion, oxytocin can be diffused through several brain areas, acting as a neuropeptide in neurochemical circuits that promote sexual behaviour, maternal and caring behaviour towards newborns, and other subtle social processes like vinculation, social memories formation, aggressiveness towards strangers and anxiety reduction. These evolutionary advantages constitute the roots for feelings of love and social phenomena like solidarity and affection. Oxytocin is increased in response to sex hormones, during pregnancy and social interactions, especially mother-child contact; additionally, is associated with endorphin release and feelings of well-being. Several studies associate the oxytocinergic system to multiple clinical implications, such as Anxiety Disorders, PTSD, Depression, Autism, Borderline and Anti-social Personality Disorder.

**Conclusions:**

Oxytocin has an important role in shaping social behaviours and in the development of secure interpersonal bonds. In the future, it can be a possible target for some psychiatric conditions; however, more research is required to prove therapeutic outcomes.

**Disclosure:**

No significant relationships.

